# Corticomotor Responses to Experimental, Acute, and Chronic Lower Limb Pain: A Systematic Review and Meta‐Analysis

**DOI:** 10.1002/brb3.70838

**Published:** 2025-09-02

**Authors:** Simon J. Summers, Jawwad Imam, Edward Gray, Ariane Suhood, Ebonie Rio, Cherylea J. Browne, Nadia Moukhaiber, Rocco Cavaleri

**Affiliations:** ^1^ Brain Stimulation and Rehabilitation (BrainStAR) Lab, School of Health Sciences Western Sydney University Western Sydney NSW Australia; ^2^ School of Biomedical Sciences Queensland University of Technology Queensland Qld Australia; ^3^ Division of Public Health, Sport and Wellbeing, Faculty of Health, Medicine and Society University of Chester Chester UK; ^4^ School of Allied Health La Trobe University Melbourne Melbourne Australia; ^5^ Translational Health and Research Institute Western Sydney University Western Sydney NSW Australia; ^6^ School of Science Western Sydney University Western Sydney NSW Australia

## Abstract

**Background**: Corticomotor adaptations are believed to contribute to persistent pain. However, prior reviews have lacked sufficient data to adequately explore these adaptations in lower limb pain. This restricts the generalizability of existing research given the distinct functional and neurophysiological differences between upper and lower limb musculature. This research gap has prompted increasing exploration of corticomotor adaptations in response to lower limb pain. Accordingly, this systematic review aimed to synthesize literature investigating corticomotor changes in response to experimental, acute clinical, and chronic lower limb pain.

**Methods**: A comprehensive search of CINAHL, Ovid MEDLINE, PubMed, Scopus, and Web of Science was conducted. Transcranial magnetic stimulation (TMS) outcomes were separated into single‐site assessments of corticomotor excitability over the motor cortical hotspot, assessments of inhibitory/facilitatory mechanisms, and corticomotor organization (mapping) outcomes. Critical appraisals were performed using the Downs and Black checklist and the TMS methodological checklist. Meta‐analyses employed random effects models.

**Results**: Analyses of 18 studies found no consistent effects of lower limb pain on motor evoked potentials. However, motor threshold data indicated that corticomotor responses may vary by region and diagnosis. Results from TMS mapping studies revealed consistent shifts in CoG for representations of painful lower limb muscles, as well as increased overlap of adjacent representations. Map volume findings differed between experimental and clinical pain, suggesting temporal variation in adaptations.

**Conclusion**: This review highlights emerging evidence that corticomotor adaptations to lower limb pain are dynamic and region‐specific. These findings lay the groundwork for future research into pain‐related motor system plasticity.

## Graphical abstract

Motor threshold varied by region and diagnosis. We also observed consistent shifts in the corticomotor representations of painful lower limb muscles. Differences in map volume between experimental and clinical pain may suggest temporal variation in corticomotor responses. Corticomotor adaptations appear dynamic, region‐specific, and influenced by pain duration.

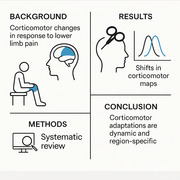

## 1 Introduction

Approximately 1.7 billion people live with chronic pain, accounting for over 6% of the global burden of disease (Arthritis and Other Musculoskeletal Conditions Across the Life Stages 2014; Mills et al. 2019). The financial cost attributed to chronic pain conditions, including direct health care expenditure and reduced productivity in the labor market, exceeds $500 billion annually in the United States alone (Ambrose and Golightly 2015). These figures are set to rise as the prevalence of chronic pain conditions, such as osteoarthritis (OA) and low back pain, continues to increase. Given the profound physical, psychological, and socioeconomic impact of chronic pain, understanding the mechanisms underlying this complex phenomenon has become a global research priority (Interagency Pain Research Coordinating Committee. 2025). Increasing emphasis has also been placed on the need to understand acute pain as a means of preventing, rather than simply managing, the development of chronic pain (Furman et al. 2019; Summers et al. 2019; Cavaleri et al. 2019).

Accumulating evidence suggests that pain drives maladaptive corticomotor changes, assessed using transcranial magnetic stimulation (TMS), that promote motor dysfunction and symptom recurrence (Hodges and Tucker 2011; Schabrun et al. 2014). A recent systematic review demonstrated a relationship between decreased corticomotor excitability in response to experimental pain (lasting minutes) and reduced pain intensity, suggesting a potentially protective early response (Chowdhury et al. 2022). However, reduced excitability was associated with *greater* pain severity in individuals with sustained experimental pain (lasting days to weeks), suggesting that persistence of this adaptation beyond the acute phase may be implicated in symptom chronicity or recurrence (Chowdhury et al. 2022).

While valuable, the data available regarding corticomotor responses to pain in previous reviews have been limited primarily to the upper limb (Chowdhury et al. 2022; Burns et al. 2016). This restricts the generalizability of existing research because changes in corticomotor excitability in response to pain may differ between upper and lower limb regions. Upper limb muscles are involved in fine motor control, while the lower limb muscles are involved in gross motor function. Further, upper limb muscle representations arise from a larger and more excitable cortical region than those of lower limb muscles (Kesar et al. 2018). Given these functional and neurophysiological differences, it has been hypothesized that the capacity for corticomotor reorganization may differ between the upper and lower limbs (Palmer and Ashby 1992; Andre‐Obadia et al. 2018; Cavaleri et al. 2023; Moukhaiber et al. 2023). The potential divergence between upper and lower limb responses is particularly pertinent given that over 70% of all musculoskeletal injuries occur in the lower limb (Murphy et al. 2003).

Previous reviews in experimental, acute clinical (< 3 months), and chronic (> 3 months) pain have all been unable to adequately probe corticomotor adaptations to lower limb pain due to a paucity of available data at the time (Chowdhury et al. 2022; Burns et al. 2016; Chang et al. 2018). The mechanisms that mediate corticomotor responses to pain (i.e., adaptations to inhibitory and facilitatory circuits) are also yet to be completely elucidated. These research gaps have prompted increasing exploration of corticomotor adaptations in response to lower limb pain, with multiple primary studies being conducted since these reviews were published. Further, no previous reviews have included data across all stages of pain, from experimental through to acute clinical and chronic. The temporal profile of corticomotor responses to lower limb pain therefore remains incompletely understood.

Thus, the aim of this systematic review was to critically appraise and synthesize existing literature investigating corticomotor changes in response to experimental, acute clinical, and chronic lower limb pain.

## 2 Method

This systematic review was conducted and written in accordance with the Preferred Reporting Items for Systematic Review and Meta‐Analyses (PRISMA) (Page et al. 2021) and A Measurement Tool to Assess Systematic Reviews (AMSTAR) (Shea et al. 2017) statements. The review protocol was prospectively registered with the International Prospective Register of Systematic Reviews (PROSPERO, registration number: CRD42020196441).

### 2.1 Eligibility Criteria

Studies were included if they (1) examined participants aged ≥ 18 years, (2) were published as full‐text studies in English, (3) were primary research, (4) compared outcomes between individuals with lower limb pain (the study had to explicitly note that participants were experiencing pain at the time of the study) and pain‐free controls, and (5) quantitatively analyzed corticomotor activity in response to lower limb pain of any type or duration using TMS. Studies were excluded if they did not include a pain‐free control group. Studies were also excluded if they assessed participants with widely distributed pain (beyond the lower limb alone), such as those with fibromyalgia. No limitations were placed upon study design as long as the above criteria were met. This broad inclusion strategy was adopted to ensure a comprehensive synthesis of available evidence examining corticomotor responses to lower limb pain.

### 2.2 Search Strategy

A comprehensive systematic search of CINAHL, Ovid MEDLINE, PubMed, Scopus, and Web of Science was conducted from inception to December 1, 2024. The specific search strategy used for these databases is presented in Table 1. The reference lists of all relevant articles were analyzed to identify additional potentially eligible studies.

**TABLE 1:** Advanced search strategy.

((Pain OR nocicept* OR discomfort OR injur* OR sore* OR strain* OR hyperalges* OR allodyn* OR “DOMS” OR aching OR ache OR tender* OR irritat* OR cramp* OR spasm* OR “NGF” OR “nerve growth factor” OR hypertonic OR capsaicin) AND (“Motor cortex” OR “M1” OR TMS OR transcranial OR corticomotor OR corticospinal) AND (Hip OR knee OR ankle OR foot OR toes OR toe OR lower limb OR thigh OR shank OR cruciate OR “ACL” OR “PCL” OR “MCL” OR medial collateral OR “LCL” OR collateral OR triad OR tibial* OR “TA” OR “RF” OR “VM” OR “VMO” OR “VL” OR menisc* OR femur OR tibia OR fibula* OR peroneal OR talus OR tarsal* OR metatars* OR ATFL OR deltoid OR patella OR quadriceps OR hamstring OR femor* OR vastus OR gastrocnemius OR soleus OR plantar OR achilles OR glute* OR semitend* OR semimem* OR groin OR acetabu* OR shin OR iliopsoas OR psoas OR iliacus OR trochant* OR Sartorius OR gracilius OR TFL OR tensor fascia latae OR pelvi* OR magnus OR adductor longus OR adductor brevis OR “PFJ” OR “PFJP” OR “PFJPS” OR “fat pad” OR calf OR plantarflex* OR dorsiflex* OR malleol*))

In addition to these systematic searches, the authors also contacted relevant experts and searched Google Scholar using derivations of “pain,” “corticomotor,” and “lower limb” for additional studies. Due to the large quantity of papers retrieved through Google Scholar searches, only the first 20 pages of results for each search were screened for relevance. Searches of the International Clinical Trials Registry Platform (ICTRP) were also conducted to identify recently completed studies.

### 2.3 Article Screening

All retrieved studies were exported to Covidence, a citation management tool (Veritas Health Innovation Ltd, VIC, Australia). Following automatic duplicate removal, two reviewers independently screened articles by title and abstract (each article was screened twice). This was followed by full‐text screening by the same authors for adherence to the eligibility criteria. Any conflicts regarding the eligibility of articles were resolved through discussion with the research team.

### 2.4 Risk of Bias Assessment

Methodological quality and risk of bias were assessed using a modified Downs and Black appraisal tool (Downs and Black 1998), which can be used for randomized and non‐randomized studies. Nine items from the appraisal tool pertaining to internal and external validity were used to assess the eligible studies. These items assessed the appropriateness of the study design, the methods underpinning participant recruitment and representativeness, assessor blinding, and data analysis. A 10th item, “Was a sample size calculation performed?” was also added to the checklist to determine whether the study had sufficient power to detect meaningful effects (Cavaleri et al. 2018). One point was allocated to each item that was fulfilled, giving a final score out of 10. Low, moderate, and high‐quality evidence were taken as follows: low: score = 0–3, moderate: score = 4–6, high: score = 7–10. Appraisals were performed by two independent reviewers. The level of agreement was quantified as a percentage, with any disagreements being resolved via discussion. Where consensus could not be reached through discussion, a third independent reviewer was consulted.

Each study was then further appraised using the TMS methodological quality checklist developed by Chipchase et al. (2012). To ensure all items were relevant and did not overlap with the Downs and Black appraisal, only the 20 items from the “Methodological Factors” section were assessed. A percentage score for reported items and controlled items was determined separately by dividing the number of satisfied items by the total number of applicable items for both categories.

### 2.5 Data Extraction

Two independent reviewers extracted the following data using a bespoke Excel data extraction form: (1) study characteristics (study design, sample size), (2) participant demographics (age, sex, diagnosis, pain intensity, pain duration), (3) TMS methodology (e.g., stimulation location, stimulation intensity, coil shape), (4) pain profile (pain intensity, duration, location, cause), and (5) corticomotor assessment outcomes (MEP amplitude, short‐interval intracortical inhibition, cortical silent period, etc.).

For crossover trials, we extracted data from the pain and control conditions within the same participants when appropriate. For parallel group studies (randomized or not), we extracted data comparing pain‐affected groups to pain‐free control groups. Where multiple time points or conditions were reported, we selected the time point or condition most representative of active pain and consistent across groups. All data were extracted as group‐level means and standard deviations, with conversion to a consistent directionality to facilitate comparison.

Disagreements were resolved via discussion or, if required, consultation with a third reviewer. Where studies did not report data in sufficient detail, authors were contacted via email to request additional information. If no response was received within two weeks, another reminder email was sent. If there was no response to the second email, ImageJ, a Java‐based image processing program, was used to extract and estimate specific data points from figures when available. If this was not possible, the data were considered irretrievable.

### 2.6 Data Analysis

TMS outcome measures were separated into single‐pulse measures of corticomotor excitability, paired‐pulse assessments of inhibitory/facilitatory mechanisms, and measures of corticomotor organization (mapping measures) (Table 2). Single‐pulse measures included motor‐evoked potential (MEP) amplitude, latency, MEP/maximal response (MEP/M‐response), resting motor threshold (rMT), active motor threshold (aMT), and cortical silent period (cSP). Paired‐pulse measures of inhibitory/facilitatory mechanisms included short‐interval intracortical inhibition (SICI), long‐interval intracortical inhibition (LICI), and intracortical facilitation (ICF). Mapping measures included map volume, map area, center of gravity (CoG), and the number of discrete peaks within tested corticomotor representations.

**TABLE 2:** Summary of eligible transcranial magnetic stimulation assessments.

Measurement type	Outcome of interest	Abbreviation
Single‐pulse measures	Resting motor threshold	rMT
	Active motor threshold	aMT
	Motor‐evoked potential amplitude	MEP amplitude
	Motor‐evoked potential/maximal response	MEP/M
	Motor‐evoked potential latency	MEP latency
	Cortical silent period	cSP
Paired‐pulse measures	Short‐interval intracortical inhibition	SICI
	Long‐interval intracortical inhibition	LICI
	Intracortical facilitation	ICF
Mapping measures	Spatial volume of the corticomotor representation	Map volume
	Area of the corticomotor representation	Map area
	Amplitude‐weighted center of the corticomotor representation (center of gravity)	CoG
	Number of discrete cortical peaks	Cortical peaks

If sufficient data (from at least two studies) were available, mean differences (MD) and 95% confidence intervals were calculated using a random effects model with Review Manager (RevMan 5, Cochrane Collaboration) software. A *p*‐value of < 0.05 was deemed statistically significant. A random‐effects model was used, as methodological heterogeneity is inevitable in TMS studies conducted across different laboratories. The impact of heterogeneity was calculated using the *I* ^2^ statistic, interpreted as follows: 0–40% may be unimportant; 30–60% may represent moderate heterogeneity; 50–90% may represent substantial heterogeneity; and 75%–100% represents considerable heterogeneity (Higgins and Green 2020).

Where possible, subgroup analyses were performed based upon pain distribution (unilateral or bilateral), pain type (experimental, clinical), and diagnosis.

To explore whether methodological quality was associated with the likelihood of reporting significant findings, we conducted a contingency table analysis at the outcome level. Each outcome assessed within a study was coded as either demonstrating significant between‐group differences or a non‐significant findings based on the author's statistical reporting. A 2 × 2 contingency table was constructed, with rows representing study quality (high vs. low or moderate) and columns representing outcome significance. A Fisher's Exact Test was used to assess whether the proportion of significant findings differed between high‐ and low‐quality studies. We did not assess publication bias via funnel plots or Egger's test, as each meta‐analysis included fewer than ten studies (Higgins and Green 2020).

## 3 Results

### 3.1 Search Results

**FIGURE 1:**
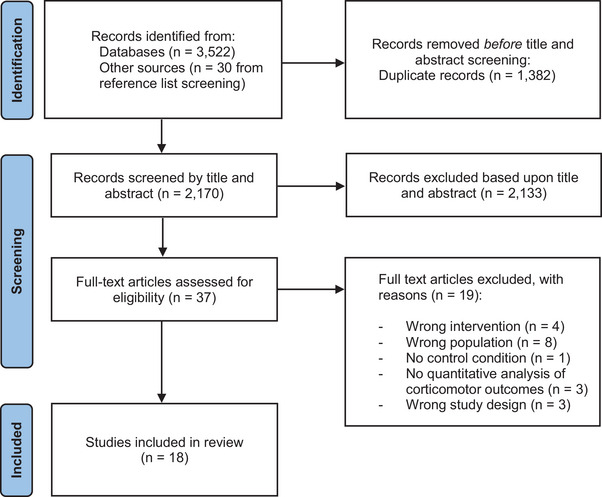
Flow of studies through review.

The initial search identified 3522 records from databases and 30 from reference list screening. Following duplicate removal (*n* = 1382), 2170 articles underwent title and abstract screening. Full‐text screening was performed on 37 articles, of which 19 were excluded (see Figure 1). This left a total of 18 studies that met the eligibility criteria for the review.

### 3.2 Study Characteristics

The characteristics of the included studies (total *n* participants = 486) are summarized in Table 3. Seven studies investigated corticomotor outcomes in response to experimental pain (106 people with pain, 80 controls), one study examined acute clinical pain (< 3 months), while the remaining ten studies investigated outcomes in chronic pain populations (146 people with pain, 118 controls). Clinical populations included people with knee OA (4 studies), pain following anterior cruciate ligament (ACL) reconstruction (1 study), patellofemoral pain syndrome (2 studies), knee pain (2 studies), patellar tendinopathy (1 study), sciatica (1 study), and ACL injury (1 study).

**TABLE 3:** **Characteristics of included studies**. Electromyography data collected from painful limb unless otherwise indicated.

Authors	Study design	Source of pain	Pain distributions	*n* (pain)	*n* (pain‐free)	EMG electrode location	Muscle contraction
Aboodarda et al. (2019)	Randomized crossover	Blood flow occlusion (thigh of non‐dominant side)	Unilateral	12	12	RF, VL, BF (dominant side)	Active
Azevedo et al. (2022)	Randomized crossover	Blood flow occlusion (thigh)	Unilateral	13	13	Pain‐free VL, RF	Active
Billot et al. (2018)	Non‐randomized crossover	Cutaneous heat over TA	Unilateral	15	15	TA	Active and resting
Caumo et al. (2016)	Case‐control	Knee OA	Unclear	27	14	Pain‐free FDI	Resting
Cavaleri et al. (2023)	RCT	Hamstring DOMS	Unilateral	26	10	BF	Active
Chipchase et al. (2018)	Case study with control	Knee OA	Unilateral	1	1	VL, VM, RF	Active
Kittelson et al. (2014)	Case‐control	Knee OA	Unclear	17	20	VL	Resting
Lepley et al. (2019)	Case‐control	ACLR	Unilateral	11	11	RF	Active
On et al. (2004)	Case‐control	PFPS	Unilateral	13	13	VMO, VL, pain‐free EDB	Resting
Rice et al. (2015)	Non‐randomized controlled trial	Hypertonic saline injection (infrapatellar fat pad)	Unilateral	18	8	VL, VM	Resting
Rio et al. (2016)	Case‐control	PT	Unilateral	11	8	RF	Active
		AKP	Unilateral	10	8		
Rittig‐Rasmussen et al. (2014)	Baseline data from experimental trial	Knee pain	Mixed	15	15	Pain‐free trapezius, APB	Active and resting
Strutton et al. (2003)	Case‐control	Sciatica	Unilateral	9	7	TA, lateral GN	Active and resting
Suhood et al. (2023)	RCT	Hypertonic saline injection (hamstring)	Unilateral	14	14	BF (bilateral)	Active
Tarrago et al. (2016)	Case‐control	Knee OA	Unilateral	21	10	Pain‐free FDI	Resting
Te et al. (2017)	Case‐control	PFPS	Bilateral	11	11	RF, VL, VM	Active
Ward et al. (2016)	Case‐control	ACL injury	Unilateral	18	18	RF	Active
Zhang et al. (2023)	Randomized crossover	Intermittent blood flow occlusion (thigh of non‐dominant side)	Unilateral	8	8	RF, VL, BF (dominant side)	Active

Abbreviations: ACLR = anterior cruciate ligament reconstruction, AKP = anterior knee pain, APB = abductor pollicis brevis, BF = biceps femoris, DOMS = delayed onset muscle soreness, EDB = extensor digitorum brevis, EMG = electromyography, FDI = first dorsal interossei, FM = fibromyalgia, GN = gastrocnemius, n = sample size, OA = osteoarthritis, PFPS = patellofemoral pain syndrome, PT = patellar tendinopathy, RCT = randomized controlled trial, RF = rectus femoris, TA = tibialis anterior, VL = vastus lateralis,, VM = vastus medialis, VMO = vastus medialis obliques.

Pain distribution differed across studies. As shown in Table 3, most studies included participants with pain in one lower limb (14 studies), one study assessed people with bilateral lower limb pain, and three studies either did not specify pain distribution or assessed a combination of individuals with unilateral and bilateral pain.

Fifteen studies measured corticomotor outcomes from lower limb muscles (rectus femoris [*n* = 8], vastus lateralis [*n* = 8], vastus medialis (including vastus medialis obliques) [*n* = 4], tibialis anterior [*n* = 2], biceps femoris [*n* = 2], and gastrocnemius [*n* = 1]). Four measured outcomes from upper body muscles in response to lower limb pain (first dorsal interossei [*n* = 2], extensor digitorum brevis [*n* = 1], abductor pollicis brevis [*n* = 1], and trapezius [*n* = 1]). Thirteen studies included data collected from muscles in a contracted (“active”) state, while eight studies included data from muscles in a relaxed (‘resting’) state.

As shown in Table 4, single‐pulse analyses included rMT (3 studies), aMT (6 studies), MEP amplitude (9 studies), MEP/M response (3 studies), MEP latency (1 study), and cSP (6 studies). Paired‐pulse analyses included ICF (2 studies), SICI (7 studies), and LICI (1 study). Five studies collected TMS mapping data, including map volume (4 studies), map area (3 studies), CoG (5 studies), and discrete peaks (1 study).

**TABLE 4:** Outcome measures assessed in included studies.

Study	rMT	aMT	MEP A	MEP/M	MEP L	cSP	SICI	LICI	ICF	Map volume	Map area	CoG	Discrete peaks
Aboodarda et al. (2019)				✓		✓							
Azevedo et al. (2022)				✓		✓	✓						
Billot et al. (2018)			✓										
Caumo et al. (2016)			✓			✓	✓						
Cavaleri et al. (2023)										✓	✓	✓	
Chipchase et al. (2018)										✓		✓	
Kittelson et al. (2014)	✓		✓				✓		✓				
Lepley et al. (2019)		✓	✓										
On et al. (2004)			✓										
Rice et al. (2015)			✓				✓						
Rio et al. (2016)		✓											
Rittig‐Rasmussen et al. (2014)		✓	✓		✓								
Strutton et al. (2003)	✓	✓			✓								
Suhood et al. (2023)										✓	✓	✓	
Tarrago et al. (2016)	✓		✓			✓	✓		✓				
Te et al. (2017)		✓‡								✓		✓	✓
Ward et al. (2016)		✓	✓			✓	✓	✓†			✓	✓	
Zhang et al. (2023)				✓		✓	✓						

Key: ✓ = assessed, † = measured with two suprathreshold pulses at 110% AMT with a 100 ms inter‐stimulus interval, ‡ = for rectus femoris only.

Abbreviations: aMT = active motor threshold, CoG = center of gravity, cSP = cortical silent period, ICF = intracortical facilitation, LICI = long interval intracortical inhibition, MEP A = motor evoked potential amplitude, MEP L = motor evoked potential latency, MEP/M = motor evoked potential/maximal response, rMT = resting motor threshold, SICI = short interval intracortical inhibition.

Twelve studies measured corticomotor outcomes from muscles that were in pain, while eight studies examined muscles remote to the source of pain. Of those eight studies, two measured outcomes from the first dorsal interossei muscle in people with knee osteoarthritis, one measured outcomes from the trapezius and abductor pollicis brevis muscles in people with knee pain, one measured outcomes from the extensor digitorum brevis muscle in people with patellofemoral pain syndrome, three measured outcomes from the rectus femoris and vastus lateralis muscles of the non‐painful limb, and one measured outcomes from the biceps femoris of the non‐painful limb (Table 3).

### 3.3 Participant Characteristics

Participant characteristics for each study are summarized in Table 5. Data were available for 486 participants. Mean participant age ranged from 21 to 66 for people in pain and 22 to 66 for controls. Pain intensity ranged from 1.4/10 to 9.8/10, and pain duration ranged from short‐lived experimental (1.8 s) to chronic (90 months).

**TABLE 5:** Key participant characteristics.

Study	Age, years (Pain)	Age, years (Control)	Sex, M/F (Pain)	Sex, M/F (Control)	Pain intensity /10	Pain duration
Aboodarda et al. (2019)	27 (4)	27 (4)	12/0	12/0	9.3 (1.1)	460 (158) s
Azevedo et al. (2022)	25 (6)	25 (5)	13/0	13/0	9.8 (0.5) cycling leg	6.9 (2.7) min
Billot et al. (2018)	25 (5)	25 (5)	6/9	6/9	3.9 (1.8) rest 3.6 (1.8) active	1.8 s
Caumo et al. (2016)	64 (8)	42 (11)	0/27	0/14	6.3 (2.2)	NR
Cavaleri et al. (2023)	22 (4)	23 (4)	11/15	5/5	5.4 (2.5)	48 h
Chipchase et al. (2018)	66	66	0/1	0/1	3.4 (0.7)	NR
Kittelson et al. (2014)	64 (7)	58 (11)	8/9	10/10	NR	NR
Lepley et al. (2019)	23 (2)	23 (2)	5/6	5/6	NR (mean KOOS = 90.6)	69.4 (22.4) months
On et al. (2004)	25 (8)	25 (7)	0/13	0/13	NR	3.46 (1.9) years
Rice et al. (2015)	29 (8)	22 (8)	7/11	2/6	5 (2.1)	20 (5.9) min
Rio et al. (2016)	26 (18‐37)* PT 26.5 (18‐37)* AKP	26 (18‐37)*	10/1 PT 6/4 AKP	7/1	5.4 (2.0) PT 5.0 (2.4) AKP	90 months (5‐192)* 90 months (12‐264)*
Rittig‐Rasmussen et al. (2014)	27 (6)	25 (3.5)	10/5	12/3	1.5 (6)	> 3 months
Strutton et al. (2003)	NR	NR	NR	NR	NR	NR
Suhood et al. (2023)	25.9 (5.2)	25.6 (5.7)	7/7	7/7	5.7 (1.9)	12.4 (5.3) minutes
Tarrago et al. (2016)	65 (8)	34 (12)	0/21	0/10	NR (mean WOMAC = 14.5)	6.73 (2.5) years
Te et al. (2017)	21 (7)	24 (6)	3/8	3/8	2.3 (2.2)	29 (26) months
Ward et al. (2016)	30 (8)	29 (7)	12/6	12/6	1.4 (1.7)	69.5 (42.5) days
Zhang et al. (2023)	25.5 (6.4)	25.5 (6.4)	4/4	4/4	6.3 (2.6)	18.9 (15.1) minutes

Green = Experimental pain, Yellow = Acute clinical pain, Red = Chronic clinical pain.

Data presented as mean (SD = standard deviation) unless otherwise indicated. Key: * = mean (SD) unavailable so data presented as median (range), — = not available, † = median, †† = all results pooled for tibialis anterior and gastrocnemius muscles.

Abbreviations: AKP = anterior knee pain, F = female, KOOS = Knee Osteoarthritis Outcome Score (pain domain), M = males, PT = patellar tendinopathy, NR = not reported or could not be calculated, , WOMAC = Western Ontario and McMaster Universities Osteoarthritis Index (pain domain).

### 3.4 Methodological Quality (Risk of Bias)

There was 99% agreement between reviewers following independent methodological appraisal using the Downs and Black (1998) checklist. Two disagreements regarding scoring were resolved via discussion without the need to consult a third reviewer. The overall methodological quality of the studies was moderate to high, with a mean Downs and Blacks score of 7.4 out of 10 and a range from 5 to10 out of 10 (Table 6). However, key limitations were identified. Most notably, only a minority of studies implemented assessor blinding, increasing the risk of detection bias. Fewer than half of the studies reported a priori sample size calculations, limiting confidence in the statistical power of their findings.

**TABLE 6:** Downs and Black quality appraisal.

Study	1	2	3	4	5	6	7	8	9	10	Total (/10)
Aboodarda et al. (2019)	✓	X	X	✓	✓	✓	✓	✓	✓	X	7
Azevedo et al. (2022)	✓	X	X	✓	✓	✓	✓	✓	✓	✓	8
Billot et al. (2018)	✓	X	X	✓	✓	✓	✓	✓	—	X	6
Caumo et al. (2016)	✓	✓	X	✓	—	✓	✓	✓	X	✓	7
Cavaleri et al. (2023)	✓	X	✓	✓	✓	✓	✓	✓	✓	X	8
Chipchase et al. (2018)	✓	X	X	✓	✓	✓	✓	✓	—	X	6
Kittelson et al. (2014)	✓	✓	X	✓	✓	✓	✓	✓	—	X	7
Lepley et al. (2019)	✓	✓	X	✓	✓	✓	✓	✓	—	X	7
On et al. (2004)	✓	✓	X	✓	✓	✓	✓	✓	—	X	7
Rice et al. (2015)	✓	X	X	✓	✓	✓	✓	✓	—	X	6
Rio et al. (2016)	✓	✓	✓	✓	✓	✓	✓	✓	✓	✓	10
Rittig‐Rasmussen et al. (2014)	✓	✓	X	✓	✓	✓	✓	✓	—	✓	8
Strutton et al. (2003)	X	✓	X	✓	—	✓	✓	✓	—	X	5
Suhood et al. (2023)	✓	X	✓	✓	✓	✓	✓	✓	✓	X	8
Tarrago et al. (2016)	✓	✓	X	✓	✓	✓	✓	✓	✓	✓	9
Te et al. (2017)	✓	✓	X	✓	✓	✓	✓	✓	✓	✓	9
Ward et al. (2016)	✓	✓	X	✓	✓	✓	✓	✓	—	X	8
Zhang et al. (2023)	✓	X	X	✓	✓	✓	✓	✓	✓	X	7

Key: ✓ = meets criteria, X = does not meet criteria, — = unable to determine.

Checklist items: 1 = Are the characteristics of the patients included in the study clearly described?, 2 = Were the subjects asked to participate in the study representative of the entire population?, 3 = Was an attempt made to blind assessors and, where possible, participants?, 4 = If any of the results of the study were based on “data dredging,” was this made clear?, 5 = In trials and cohort studies, do the analyses adjust for different lengths of follow‐up of participants, or in case‐control studies, is the time period between the intervention and outcome the same for cases and controls?, 6 = Were the statistical tests used to assess the main outcomes appropriate?, 7 = Were the main outcome measures used accurate (valid and reliable)?, 8 = Were losses of participants to follow‐up taken into account?, 9 = Were participants in each group recruited over the same period of time?, 10 = Was a sample size calculation performed?.

No disagreements occurred between reviewers during further appraisal using the TMS methodological checklist developed by Chipchase et al. (2012). The results of this appraisal are presented in Table 7. Overall, the mean scores were 73% for reported items and 68% for controlled items. Items that were reported or controlled by less than half of the included studies were “prior activity of the muscle to be tested,” “level of relaxation of muscles other than those being tested,” “direction of induced current in the brain,” “pulse shape,” and “subject attention (level of arousal) during testing.” These methodological limitations reduce the internal validity and comparability of results and should be addressed in future trials to enhance rigor and reproducibility.

**TABLE 7:** TMS checklist.

Item		Aboodarda et al. (2019)	Azevedo et al. (2022)	Billot et al. (2018)	Caumo et al. (2016)	Cavaleri et al. (2023)	Chipchase et al. (2018)	Kittelson et al. (2014)	Lepley et al. (2019)	On et al. (2004)	Rice et al. (2015)	Rio et al. (2016)	Rittig‐Rasmussen et al. (2014)	Strutton et al. (2003)	Suhood et al. (2023)	Tarrago et al. (2016)	Te et al. (2017)	Ward et al. (2016)	Zhang et al. (2023)
1	Reported	✓	✓	✓	✓	✓	✓	✓	✓	✓	✓	✓	✓	✓	✓	✓	✓	✓	✓
	Controlled	✓	✓	✓	✓	✓	✓	✓	✓	✓	✓	✓	✓	X	✓	✓	✓	✓	✓
2	Reported	✓	✓	✓	✓	✓	✓	✓	✓	✓	X	✓	✓	✓	✓	✓	✓	✓	✓
	Controlled	✓	✓	✓	✓	✓	✓	✓	✓	✓	X	✓	X	✓	✓	✓	✓	✓	✓
3	Reported	✓	X	X	X	✓	X	X	X	X	X	✓	✓	X	✓	X	X	X	✓
	Controlled	✓	X	X	X	✓	X	X	X	X	X	X	X	X	✓	X	X	X	✓
4	Reported	NA	NA	NA	NA	NA	NA	NA	NA	NA	NA	NA	NA	NA	NA	NA	NA	NA	NA
	Controlled	✓	X	X	X	✓	X	X	X	X	X	X	X	X	✓	X	X	X	X
5	Reported	✓	✓	✓	✓	✓	✓	✓	✓	✓	✓	✓	X	✓	✓	X	✓	✓	✓
	Controlled	✓	✓	✓	✓	✓	✓	✓	✓	✓	✓	✓	✓	✓	✓	✓	✓	✓	✓
6	Reported	X	X	✓	✓	✓	✓	✓	X	✓	X	X	✓	✓	✓	X	✓	X	X
	Controlled	X	X	✓	✓	✓	✓	✓	X	✓	X	X	✓	✓	✓	X	✓	X	X
7	Reported	✓	X	X	X	✓	✓	X	X	X	✓	✓	X	✓	✓	X	✓	X	X
	Controlled	✓	X	X	X	✓	✓	X	X	X	✓	✓	X	✓	✓	X	✓	X	X
8	Reported	X	X	✓	X	✓	✓	✓	✓	X	✓	X	X	X	✓	✓	✓	✓	✓
	Controlled	X	X	✓	X	✓	✓	✓	✓	X	✓	X	X	X	✓	✓	✓	✓	✓
9	Reported	✓	✓	✓	✓	✓	✓	✓	✓	✓	✓	✓	✓	✓	✓	✓	✓	✓	✓
	Controlled	✓	✓	✓	✓	✓	✓	✓	✓	✓	✓	✓	✓	✓	✓	✓	✓	✓	✓
10	Reported	✓	✓	✓	✓	✓	✓	✓	✓	✓	✓	✓	✓	✓	✓	✓	✓	✓	✓
	Controlled	✓	✓	✓	✓	✓	✓	✓	✓	✓	✓	✓	X	✓	✓	✓	✓	✓	✓
11	Reported	X	X	X	X	✓	X	X	X	X	X	X	X	X	✓	X	X	✓	X
	Controlled	X	X	X	X	✓	X	X	X	X	X	X	X	X	✓	X	X	✓	X
12	Reported	✓	✓	✓	X	✓	X	✓	✓	✓	✓	✓	✓	X	✓	✓	X	✓	✓
	Controlled	✓	✓	✓	X	✓	X	✓	✓	✓	✓	✓	✓	X	✓	✓	X	✓	✓
13	Reported	✓	✓	✓	✓	✓	✓	✓	X	X	✓	✓	✓	X	✓	✓	X	✓	✓
	Controlled	✓	✓	✓	✓	✓	✓	✓	X	X	✓	X	X	X	✓	✓	X	✓	✓
14	Reported	X	✓	✓	NA	✓	✓	NA	NA	NA	NA	NA	✓	NA	NA	NA	NA	NA	✓
	Controlled	X	X	✓	NA	✓	✓	NA	NA	NA	NA	NA	✓	NA	NA	NA	NA	NA	✓
15	Reported	✓	X	✓	X	✓	✓	X	X	X	X	X	X	✓	✓	X	X	✓	✓
	Controlled	✓	X	✓	X	✓	✓	X	X	X	X	X	X	✓	✓	X	X	✓	✓
16	Reported	✓	✓	✓	✓	✓	✓	✓	✓	✓	✓	✓	✓	✓	✓	✓	✓	✓	✓
	Controlled	✓	✓	✓	✓	✓	✓	✓	✓	✓	✓	✓	✓	✓	✓	✓	✓	✓	✓
17	Reported	✓	X	✓	✓	✓	X	✓	✓	✓	X	✓	✓	✓	✓	✓	✓	✓	✓
	Controlled	✓	X	✓	✓	✓	X	✓	✓	✓	X	✓	✓	✓	✓	✓	✓	✓	✓
18	Reported	NA	NA	NA	✓	NA	NA	✓	NA	NA	✓	NA	NA	NA	NA	NA	NA	✓	✓
	Controlled	NA	NA	NA	✓	NA	NA	✓	NA	NA	✓	NA	NA	NA	NA	NA	NA	✓	✓
19	Reported	NA	NA	NA	✓	NA	NA	✓	NA	NA	✓	NA	NA	NA	NA	NA	NA	✓	✓
	Controlled	NA	NA	NA	✓	NA	NA	✓	NA	NA	✓	NA	NA	NA	NA	NA	NA	✓	✓
20	Reported	NA	NA	NA	✓	NA	NA	✓	NA	NA	✓	NA	NA	NA	NA	NA	NA	✓	✓
	Controlled	NA	NA	NA	✓	NA	NA	✓	NA	NA	✓	NA	NA	NA	NA	NA	NA	✓	✓
	% Reported	75	56	81	67	100	75	78	60	60	67	73	69	67	100	60	67	83	84
	% Controlled	76	47	76	63	100	71	74	56	56	63	56	47	56	100	63	63	79	80

Key: ✓ = Yes, X = No,

Abbreviation: NA = not applicable.

There was no significant effect of study quality on the likelihood of reporting a significant finding (odds ratio = 0.525, *p* = 0.47).

### 3.5 Effect of Lower Limb Pain on Corticomotor Measures From the Painful Limb

#### 3.5.1 Single‐pulse Measures of Corticomotor Excitability

##### 3.5.1.1 Resting Motor Threshold

**FIGURE 2:**
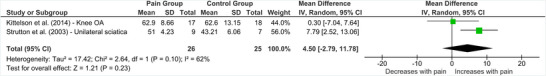
**Effect of lower limb pain on resting motor threshold**. Abbreviations: CI = confidence interval, IV = inverse variance, OA = osteoarthritis**, SD = standard deviation.

Two studies investigated the effect of lower limb pain on rMT collected over the representation of the painful region (Kittelson et al. 2014; Strutton et al. 2003). Both studies were conducted in chronic lower limb pain populations, with no studies of experimental or acute clinical pain assessing this outcome. As shown in Figure 2, the overall pooled mean difference demonstrated no significant change in rMT among individuals with lower limb pain compared to healthy controls (MD = 4.50 [95% CI, ‐2.79 to 11.78], *p* = 0.23, *I* ^2^ = 62%). There were insufficient data available to facilitate further subgrouping for this outcome.

### 3.6 Active Motor Threshold

**FIGURE 3:**
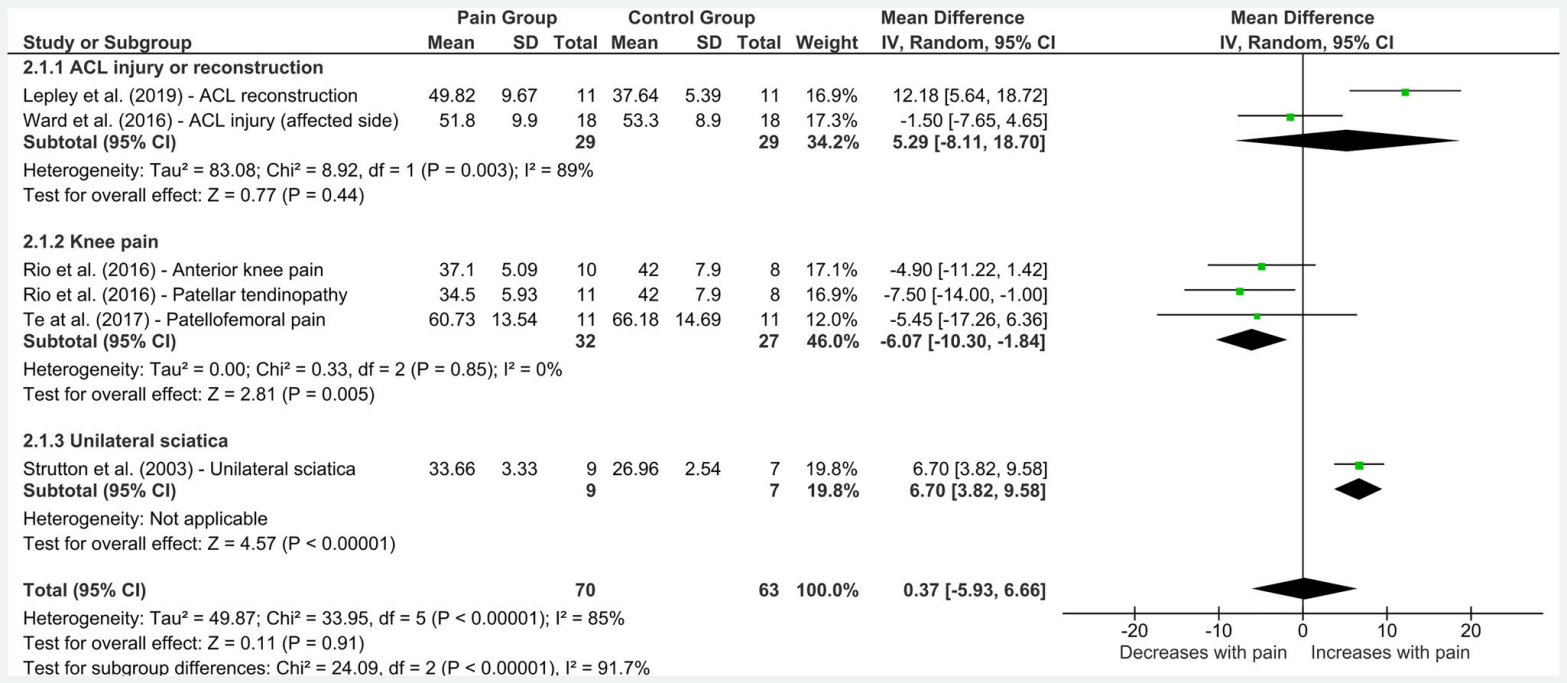
**Effect of lower limb pain on active motor threshold**. Abbreviations: ACL = anterior cruciate ligament, CI = confidence interval, IV = inverse variance, SD = standard deviation.

Six studies explored the influence of lower limb pain on aMT collected over the representation of the painful region (Strutton et al. 2003; Lepley et al. 2019; Rio et al. 2016; Te et al. 2017; Ward et al. 2016). One study investigated acute clinical pain (Ward et al. 2016), while the remaining studies explored chronic pain. As shown in Figure 3, no overall difference in aMT was observed between people with and without lower limb pain (MD = 0.37 [95% CI = ‐5.93 to 6.66], *p* = 0.91). However, there was substantial heterogeneity in this pooled effect estimate (*I* ^2^ = 85%). Limiting the analysis to studies of knee pain reduced heterogeneity (*I* ^2^ = 0%) and revealed a significant reduction in aMT among this population compared to controls (MD = ‐6.07 [95% CI = ‐10.30 to ‐1.84], *p* < 0.01). Including only studies of ACL injury or reconstruction in the analysis revealed no differences in aMT between those within and without pain (Figure 3). One study exploring unilateral sciatica revealed a significant increase in aMT (MD = 6.70 [95% CI = 3.82 to 9.58], *p* < 0.01).

#### 3.6.1 Motor‐evoked Potential Amplitude

**FIGURE 4:**
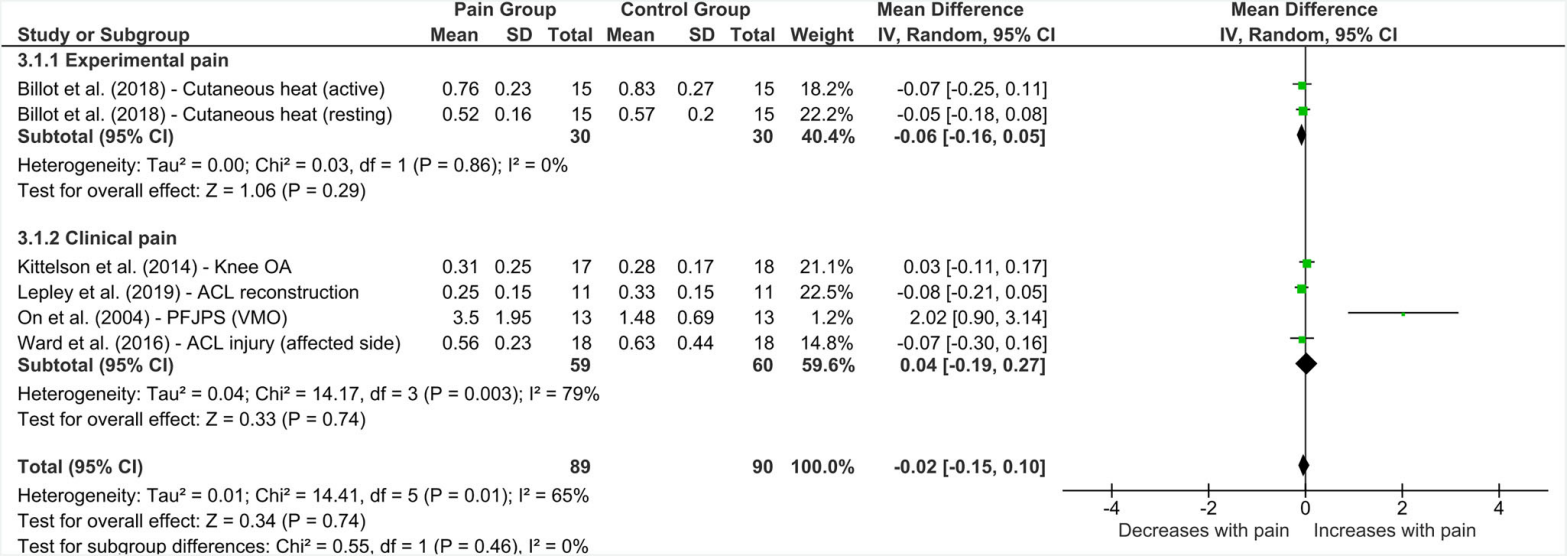
**Effect of lower limb pain on motor evoked potential amplitude**. Abbreviations: ACL = anterior cruciate ligament, CI = confidence interval, EDB = extensor digitorum brevis, IV = Inverse variance, OA = osteoarthritis, PFJPS = patellofemoral joint pain syndrome, SD = standard deviation, VMO = vastus medialis oblique.

Six studies investigated the influence of lower limb pain on MEP amplitude collected over the representation of the painful area (Kittelson et al. 2014; Lepley et al. 2019; Ward et al. 2016; Billot et al. 2018; On et al. 2004; Rice et al. 2015). Of these studies, two involved people with experimentally induced pain, one involved people with acute clinical pain, and three involved participants with chronic lower limb pain (Figure 4). One study (Rice et al. 2015) reported significant increases in MEP amplitudes recorded from vastus lateralis and vastus medialis during pain induced via injection of hypertonic saline into the infrapatellar fat pad. However, this study did not report unnormalized MEP data, and as the relevant data could not be retrieved from the authors, the study was excluded from the meta‐analysis. This left five studies to be included.

Overall, there were no differences between people with and without lower limb pain in terms of MEP amplitude (MD = ‐0.02 [95% CI = ‐0.15 to 0.10], *p* = 0.74, *I* ^2^ = 65%). As shown in Figure 4, subgrouping by pain type (experimental, clinical) revealed no significant between‐group difference in MEP amplitudes. There were insufficient data available to facilitate further subgrouping for this outcome.

#### 3.6.2 Motor‐evoked Potential Latency

One study (Strutton et al. 2003) reported that there were no significant differences in MEP latency between people with and without unilateral sciatica but did not provide sufficient data for further analysis.

### 3.7 Cortical Silent Period

One study investigated the influence of lower limb pain on cSP measures collected over the representation of the painful area in people with acute ACL injury (Ward et al. 2016). The study reported no significant difference between individuals with lower limb pain and those without pain in terms of cSP (MD = 13.00 [95% CI ‐6.93 to 32.93], *p* = 0.20).

### 3.8 Paired‐pulse Measures of Inhibitory/Facilitatory Mechanisms

#### 3.8.1 Short‐interval Intracortical Inhibition

**FIGURE 5:**
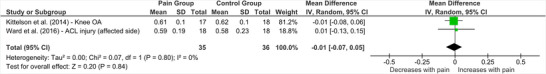
**Effect of lower limb pain on short‐interval intracortical inhibition**. Abbreviations: ACL = anterior cruciate ligament, CI = confidence interval, IV = inverse variance, OA = osteoarthritis, SD = standard deviation.

As shown in Figure 5, two studies explored the influence of lower limb pain on SICI measures collected over the representation of the painful area (Kittelson et al. 2014; Ward et al. 2016). Overall pooled effects revealed no effect of lower limb pain on SICI (MD = ‐0.01 [95% CI ‐0.07 to 0.05], *p* = 0.84, *I* ^2^ = 0%).

#### 3.8.2 Long‐interval Intracortical Inhibition

One study measured LICI in people with pain following ACL injury (mean [SD] time post‐injury of 69.5 [42.5] days) (Ward et al. 2016). No significant difference in LICI was observed for people with (mean [SD] = 0.59 [0.22]) and without (mean [SD] = 0.55 [0.13]) lower limb pain.

#### 3.8.3 Intracortical Facilitation

One study (Kittelson et al. 2014) investigated the effects of knee OA on ICF measurements from painful lower limb muscles. No significant differences in ICF were found between those with and without pain.

### 3.9 Measures of Corticomotor Organization (TMS Mapping Outcomes)

#### 3.9.1 Map Volume

**FIGURE 6:**
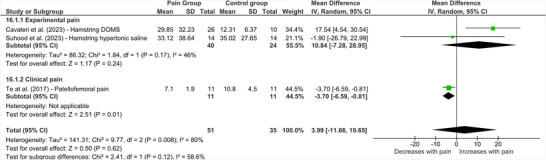
**Effect of lower limb pain on TMS map volume**. Abbreviations: CI = confidence interval, DOMS = delayed onset muscle soreness, IV = inverse variance, SD = standard deviation.

Four studies examined the influence of lower limb pain on TMS map volume derived from the representation of the painful side (Cavaleri et al. 2023; Te et al. 2017; Chipchase et al. 2018; Suhood et al. 2024). One of these studies (Chipchase et al. 2018) was a case study of a person with knee OA, who demonstrated reduced TMS map volume when compared to an age‐ and sex‐matched control. As studies with *n* = 1 do not provide estimates of population variability, the data from this study could not be included in the meta‐analysis. Accordingly, Figure 6 presents the pooled effect estimate across the three remaining studies, two of which explored the effect of experimentally induced hamstring pain on map volume recordings from hamstring muscle representations (Cavaleri et al. 2023), and the other assessed map volume recordings from quadriceps representations in people with knee OA compared to healthy controls (Te et al. 2017) (*Note*: rectus femoris data presented in Figure 6). Similar findings were also observed for vastus lateralis and vastus medialis but were removed from meta‐analysis to avoid unit‐of‐analysis errors. As shown in Figure 6, pooled estimates demonstrated no significant difference in map volume between those with and without lower limb pain (MD = 3.99 [95% CI ‐11.66 to 19.65], *p* = 0.62, *I* ^2^ = 80%). Subgrouping by pain type revealed no significant difference in map volume between those with experimental pain and healthy controls (MD = 10.84 [95% CI ‐7.28 to 28.95], *p* = 0.24, *I* ^2^ = 46%). However, one study exploring chronic patellofemoral pain demonstrated significant reductions in map volume (MD = ‐3.70 [95% CI ‐6.59 to ‐0.81], *p* = 0.01).

Two studies (Cavaleri et al. 2023; Suhood et al. 2024) also examined inter‐individual differences in corticomotor responses to experimentally induced lower limb pain. Both studies found that corticomotor depression (reduced map volume) was correlated with lower hamstring muscle sensitivity, assessed using pressure pain thresholds. Corticomotor facilitation (increased map volume) was associated with increased sensitivity. Suhood et al. (2024) also reported that corticomotor depression was associated with increased knee flexion maximal voluntary contractions (MVCs), and corticomotor facilitation was associated with reduced knee flexion MVCs.

#### 3.9.2 Map Area

**FIGURE 7:**
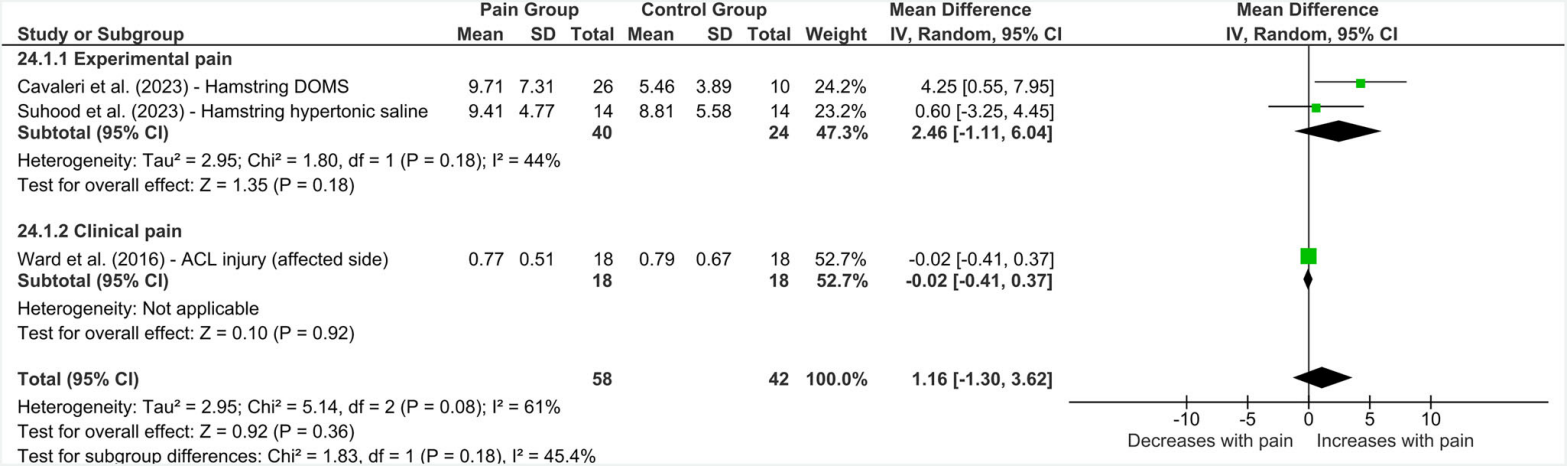
**Effect of lower limb pain on TMS map area**. Abbreviations: ACL = anterior cruciate ligament, CI = confidence interval, DOMS = delayed onset muscle soreness, IV = inverse variance, SD = standard deviation.

Three studies investigated the effect of lower limb pain on the size (area) of corticomotor representations (Cavaleri et al. 2023; Ward et al. 2016; Suhood et al. 2024). As shown in Figure 7, pooled effects demonstrated no significant differences between individuals with and without lower limb pain (MD = 1.16 [95% CI ‐1.30 to 3.62], *p* = 0.36, *I* ^2^ = 61%). Subgrouping based upon pain type (experimental or clinical) had no effect on the overall findings.

### 3.10 Center of Gravity

Five studies investigated the influence of lower limb pain on map CoG (Cavaleri et al. 2023; Te et al. 2017; Ward et al. 2016; Chipchase et al. 2018; Suhood et al. 2024). A study of experimentally induced hamstring pain (DOMS) found significant shifts in biceps femoris representation CoG, reflective of corticomotor reorganization, between groups (*t*_34_ = 2.73, *p* = 0.01) (Cavaleri et al. 2023). Specifically, the experimental group demonstrated greater CoG displacement from the pre‐pain baseline (1.2 ± 0.8) than the control group (0.4 ± 0.1). This was supported by a subsequent study of experimental hamstring pain induced via hypertonic saline injection. The study reported that CoG displacement varied between 0 and 3 cm from baseline in the experimental group for both the painful and non‐painful limbs, whilst no appreciable change was found in the control group (Suhood et al. 2024). In a study of 11 people with patellofemoral pain (and 11 age‐ and sex‐matched controls), Te et al. (2017) identified that the distance between the rectus femoris, vastus lateralis, and vastus medialis CoGs for each muscle pair was reduced in people with pain compared to healthy controls. This study also found that CoG was located more anteriorly for all three muscles in people with patellofemoral pain compared to healthy controls. Chipchase et al. (2018) also reported greater overlap of adjacent muscle representations in a participant with knee OA compared to a healthy age‐ and sex‐matched control. Conversely, Ward et al. (2016) found no difference in map CoG between individuals with acute pain related to ACL injury and healthy controls.

#### 3.10.1 Discrete Peaks

One study analyzed the number of discrete cortical peaks for the rectus femoris, vastus lateralis, and vastus medialis muscle representations in people with chronic patellofemoral pain (Te et al. 2017). This study found a decrease in the number of discrete cortical peaks across all three muscles in the lower limb pain population compared to the control group (F_1, 60_ = 7.35, *p* < 0.01).

## 4 Effect of Lower Limb Pain on Corticomotor Measures From Remote Regions and the (Non‐painful) Contralateral Limb

### 4.1 Single‐pulse Measures of Corticomotor Excitability

#### 4.1.1 Resting Motor Threshold

One study assessed rMT for muscles remote to the site of pain (MdGL et al. 2016). This study assessed rMT for the FDI muscle in people with pain related to unilateral knee OA and reported a significant increase in FDI rMT (*p* < 0.05).

### 4.2 Active Motor Threshold

**FIGURE 8:**
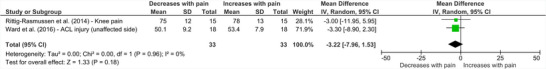
**Effect of lower limb pain on active motor threshold from remote and non‐painful representations**. Abbreviations: ACL = anterior cruciate ligament, CI = confidence interval, IV = inverse variance, SD = standard deviation.

Two studies assessed changes in aMT for muscles beyond the painful region. Rittig‐Rasmussen et al. (2010) examined the aMT of the trapezius and APB muscles in response to knee pain, and Ward et al. (2016) assessed the aMT of the rectus femoris muscle during pain from an ACL injury on the contralateral limb. As shown in Figure 8, pooling data from these two studies revealed no effects of lower limb pain on aMT taken from muscles remote to the source of pain (MD = ‐3.22 [95% CI ‐7.96 to 1.53], *p* = 0.18, *I* ^2^ = 0%).

### 4.3 Motor‐evoked Potential Amplitude

**FIGURE 9:**
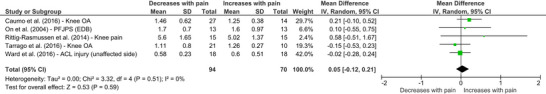
**Effect of lower limb pain on motor evoked potentials from remote and non‐painful representations**. Abbreviations: ACL = anterior cruciate ligament, CI = confidence interval, EDB = extensor digitorum brevis, IV = inverse variance, OA = osteoarthritis, PFJPS = patellofemoral joint pain syndrome, SD = standard deviation.

Five studies included data regarding changes in MEP amplitude recordings from muscles beyond the painful lower limb (Ward et al. 2016; On et al. 2004; MdGL et al. 2016; Caumo et al. 2016; Rittig‐Rasmussen et al. 2014). Of these studies, four assessed MEPs for pain‐free upper limb muscles, and one measured MEPs for lower limb muscles contralateral to the side of pain. Pooling data from these studies revealed no effects of lower limb pain on MEP amplitudes taken from remote, non‐painful muscle representations (MD = 0.05 [95% CI ‐0.12 to 0.21], *p* = 0.59, *I* ^2^ = 0%) (Figure 9). There were insufficient data available to facilitate further subgrouping for this outcome.

### 4.4 Motor‐evoked Potential / Maximal Response

Three studies measured MEP/M‐response (Azevedo et al. 2022; Zhang et al. 2023; Aboodarda et al. 2020). Each study explored the effects of experimental leg pain (blood flow occlusion) on corticospinal excitability of the contralateral (pain‐free) limb during single‐leg exercise. Pooled analysis could not be performed due to considerable methodological and reporting heterogeneity. Azevedo et al. (2022) reported no differences in MEP/M‐response recordings between individuals with and without experimentally induced pain. Similarly, Zhang et al. (2023) and Aboodarda et al. (2020) reported no significant difference in MEP/M‐response between people with and without experimental pain in the opposite lower limb.

### 4.5 Motor‐evoked Potential Latency

Rittig‐Rasmussen et al. (2014) measured MEP latency for muscles remote to the lower limb pain (trapezius and APB) and found no significant difference in MEP latency between individuals with knee pain and controls.

### 4.6 Cortical Silent Period

**FIGURE 10:**
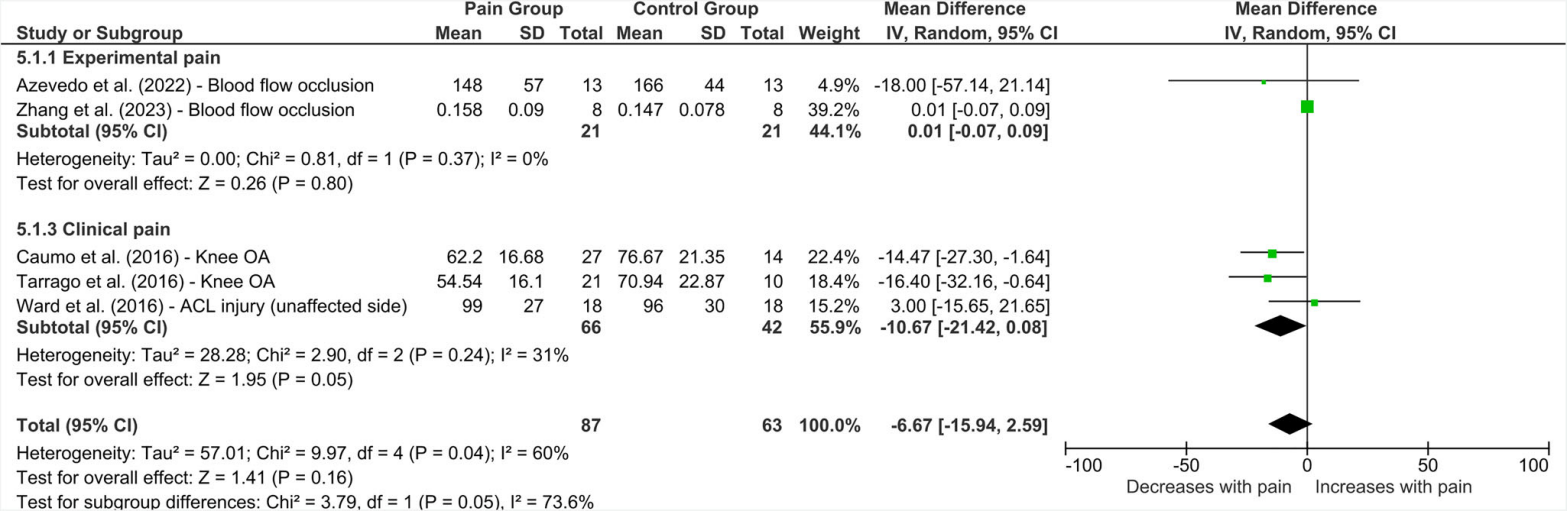
Effect of lower limb pain on cSP from remote and non‐painful representations**. Abbreviations: ACL = anterior cruciate ligament, CI = confidence interval, IV = inverse variance, OA = osteoarthritis, SD = standard deviation.

Six studies presented data regarding the influence of lower limb pain on cSP for muscle representations remote to the site of pain (Figure 10) (Ward et al. 2016; MdGL et al. 2016; Caumo et al. 2016; Azevedo et al. 2022; Zhang et al. 2023; Aboodarda et al. 2020). Aboodarda et al. (2020) did not present mean milliseconds (SD) cortical silent period data, and so their study was excluded from the analysis. Pooled effects from the remaining studies revealed no significant difference in cSP for remote muscles in people with lower limb pain compared to healthy controls (MD = ‐6.67 [95% CI ‐15.94 to 2.59], *p* = 0.16, *I* ^2^ = 60%). Subgrouping by pain type revealed no significant change in cSP among people with clinical pain or experimentally induced pain (Figure 10).

### 4.7 Short‐interval Intracortical Inhibition

**FIGURE 11:**
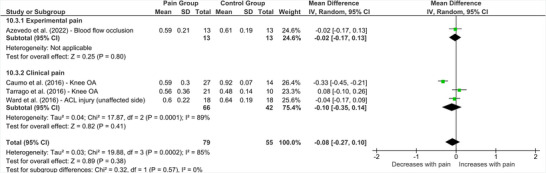
Effect of lower limb pain on SICI from remote and non‐painful representations**. Abbreviations: ACL = anterior cruciate ligament, CI = confidence interval, IV = inverse variance, OA = osteoarthritis, SD = standard deviation.

Four studies measured SICI over the representations of muscles remote to the site of pain (Figure 11). Pooled effects revealed no significant difference between SICI recordings taken from remote, pain‐free areas compared to healthy controls (MD = ‐0.08 [95% CI ‐0.27 to 0.10], *p* = 0.38, *I* ^2^ = 85%). Subgrouping by pain type (experimental or clinical) did not reveal any significant differences between people with lower limb pain and healthy controls.

### 4.8 Long‐interval Intracortical Inhibition

One study measured LICI over the representation of the non‐painful limb of people with pain following an ACL injury (Ward et al. 2016). There was no significant difference between LICI recordings taken for the uninjured limb compared to healthy controls.

### 4.9 Intracortical Facilitation

One study measured ICF measurements taken from remote upper limb muscles in people with knee OA (MdGL et al. 2016). There was no significant difference between ICF recordings from pain‐free FDI in people with lower limb pain compared to healthy controls.

### 4.10 Measures of Corticomotor Organization (TMS Mapping Outcomes)

One study assessed the effect of experimental hamstring pain on the corticomotor representation of the limb opposite to the side of pain (Suhood et al. 2024). At a group level, the study reported there were no significant changes in map volume, map area, or CoG displacement. However, in alignment with findings for the painful limb, there were significant inter‐individual differences within the experimental group, with 64% of participants exhibiting an increase in map volume (corticomotor facilitation) and 44% a decrease in map volume (corticomotor depression). Similar variability was reported in map area responses for the non‐painful limb, and the CoG displacement in the representation of the non‐painful hamstring varied between 0 and 3 cm from baseline. There were no differences in map volume, area, or CoG over time for the control group. Furthermore, the study reported that corticomotor depression was correlated with lower hamstring muscle sensitivity and increased knee flexion MVCs on the non‐painful limb. Corticomotor facilitation was associated with increased sensitivity and reduced knee flexion MVCs.

## 5 Discussion

This systematic review synthesized the available literature regarding corticomotor responses to lower limb pain, assessed using transcranial magnetic stimulation. Meta‐analyses revealed no significant difference in MEP amplitude between individuals with and without lower limb pain. However, findings from other single‐pulse assessments of corticomotor excitability (rMT, aMT) were conflicting, with pooled effects varying between diagnoses. Most assessments of inhibitory/facilitatory circuits (SICI, LICI, ICF) demonstrated no differences between individuals with and without lower limb pain. Results from TMS mapping studies revealed consistent shifts in CoG for representations of painful lower limb muscles, as well as increased overlap of adjacent representations.

This review found no difference in MEP amplitudes between individuals with and without lower limb pain. However, subgroup analyses revealed variable changes in rMT and aMT. While neuropathic pain (unilateral sciatica) was associated with increases in motor threshold, suggestive of reduced neuronal and interneuronal membrane excitability, no such changes were observed in knee OA. Conversely, chronic patellofemoral (knee) pain was associated with *reductions* in aMT, reflecting increases in corticomotor excitability. Such findings indicate that the effects of lower limb pain on corticomotor excitability may be diagnosis‐ or condition‐specific. This notion is supported by previous work suggesting that corticomotor responses to neuropathic pain may differ from those associated with non‐neuropathic or musculoskeletal pain (Barbosa et al. 2023). Another interpretation of this discrepancy could be that corticomotor responses to pain vary between muscle groups. The potential for region‐specific differences in corticomotor reorganization is supported by findings that motor training protocols induce more effective corticomotor inhibition in distal than proximal upper limb muscle representations (Barbosa et al. 2023). Further, corticomotor adaptations to motor training have been shown to differ between upper limb, lower limb, and low back muscles (Cavaleri et al. 2020; van de Ruit and Grey 2019; Beck et al. 2007; Sugawara et al. 2016). It is therefore plausible that anterior and posterior thigh representations may have distinct capacities for corticomotor reorganization. However, given the inability to subgroup by muscle for most outcomes in this review, such interpretations should be considered with a degree of caution.

The absence of pooled effects for single‐site analyses could also be due to inter‐individual variability in corticomotor responses. A recent meta‐analysis including five studies of upper limb pain found that 57% of data points taken during sustained pain reflected decreased corticomotor excitability (compared to pain‐free baseline measures) and 43% reflected increased excitability (Chowdhury et al. 2022). Such variability in corticomotor responses is consistent with that observed following motor training (Cavaleri et al. 2020; van de Ruit and Grey 2019) and non‐invasive brain stimulation protocols (Wiethoff et al. 2014; Müller‐Dahlhaus et al. 2008; Fratello et al. 2006; Martin et al. 2006). Although not completely understood, factors including prior synaptic activity, anatomical differences (e.g., cortical thickness), exercise history, and affective‐emotional state are thought to contribute to variability in corticomotor responses to pain (Ridding and Ziemann 2010; Summers et al. 2020). This inter‐individual variability could “wash out” pooled group‐level effects during meta‐analyses.

Unfortunately, the present review identified only two studies investigating inter‐individual variability in corticomotor responses to lower limb pain (Suhood et al. 2024). One study of 36 participants found that corticomotor depression was correlated with lower hamstring muscle sensitivity, suggesting a potentially protective response (Cavaleri et al. 2023). A second study of 28 participants reported similar findings but additionally reported that corticomotor depression was associated with increased knee flexion MVCs (Suhood et al. 2024). Such findings contrast with findings from upper limb studies, which have found corticomotor depression to be associated with potentially detrimental outcomes in the transition to sustained pain (Chowdhury et al. 2022; Summers et al. 2019). However, it is important to note that, while corticomotor depression was associated with improved mechanical sensitivity in the lower limb, no such relationship was observed in terms of pain intensity (on a numerical rating scale). Further work is therefore needed to better understand the variability of corticomotor responses to lower limb pain and the influence that this variability may have on patient symptoms.

While valuable, single‐site TMS analyses provide limited information on corticomotor reorganization, which encompasses changes in the size, distribution, or location of muscle representations (Rossi et al. 2009). To gain insight into these factors, TMS mapping is required. The present review demonstrates that lower limb pain is associated with shifts in the TMS map‐derived CoG of affected muscle representations. Greater overlap or ‘smudging’ of representations may promote *en masse* muscle recruitment to “splint” and protect painful regions (Hodges and Tucker 2011; Schabrun et al. 2014). Though beneficial in the short term, this loss of individuated control is hypothesized to contribute to maladaptive motor strategies and suboptimal joint loading over time, which could promote pain recurrence and chronicity (Tsao et al. 2011). One study has demonstrated that lower limb pain is also associated with decreases in the number of “discrete peaks” throughout corticomotor representations (Te et al. 2017). Multiple discrete peaks in excitability are thought to be required for inter‐muscle coordination during different activities (Massé‐Alarie et al. 2017), so reductions in the number of peaks may also contribute to the poor inter‐muscle coordination observed in chronic lower limb pain.

This review provides preliminary evidence that corticomotor responses to lower limb pain may vary over time. *Chronic* pain following ACL reconstruction (pain duration approximately 69.4 months) was associated with an increase in aMT (decreased corticomotor excitability) (Lepley et al. 2019) compared to healthy controls, while no differences were observed between controls and people with *acute* pain (pain duration approximately 69.5 days) following ACL injury (Ward et al. 2016). Similarly, experimental lower limb pain was associated with increases or no change in map volume, while chronic pain was associated with reductions in map volume. Map volume provides insight into the excitability of an entire representation, as opposed to single‐site analyses, which derive data from stimulation of the hotspot alone. Increases in map volume and reductions in aMT are thought to reflect corticomotor changes that serve to splint painful regions and reduce movement to prevent further injury (Lund et al. 1991). Conversely, reductions in map volume and increases in aMT suggest decreases in corticomotor output, potentially reflecting the adaptive strategy of “offloading” to decrease movement in the painful region (Te et al. 2017). The observed differences between experimental/acute and chronic pain in this review imply that corticomotor responses to lower limb pain may change over time, potentially presenting an opportunity to prevent pain chronicity by “normalizing” excitability during the early phases of pain. However, further research is necessary to confirm this hypothesis due to the limited number of studies exploring TMS mapping outcomes in the lower limb.

Most single‐site transcranial magnetic stimulation (TMS) studies to date have demonstrated minimal or no effects of lower limb pain on the corticomotor representation of remote, non‐painful body regions. This suggests that, in many cases, pain localized to the lower limb does not significantly alter motor cortical excitability or representation in areas unrelated to the site of pain. However, some research challenges this notion, indicating that lower limb pain may induce broader neurophysiological changes. For example, studies have reported that lower limb pain can influence resting motor threshold (rMT) in remote musculature (MdGL et al. 2016), alter TMS‐derived corticomotor maps of upper limb muscles, and affect the cortical representation of the limb contralateral to the painful site (Suhood et al. 2024). Notably, widespread corticomotor reorganization has been observed more consistently in upper limb pain conditions, such as lateral epicondylalgia and chronic shoulder pain (Chang et al. 2018), raising the possibility that pain, irrespective of its anatomical location, may induce broader motor system adaptations. Nevertheless, the extent, nature, and functional relevance of such changes in the context of lower limb pain remain poorly understood. Further research is required to delineate the mechanisms underpinning corticomotor reorganization associated with lower limb pain, including whether these effects are consistent across different pain conditions, vary with chronicity or intensity, and have meaningful consequences for motor control or rehabilitation outcomes.

This review identified several limitations with existing literature. For example, assessor blinding was performed in only two studies, and fewer than half of the included studies performed a priori sample size calculations. The lack of blinding increases the risk of detection bias, particularly for outcomes involving subjective interpretation, while inadequate sample size planning raises concerns about statistical power and the potential for false‐negative findings (Strutton et al. 2003; Cavaleri et al. 2020). Additionally, use of the TMS methodological checklist developed by Chipchase et al. (2012) was limited (though it is acknowledged that a small number of studies were developed before this checklist was created), with important factors such as subject attention either not controlled or unreported across most studies. Further, while there is accumulating cross‐sectional research exploring the influence of chronic lower limb pain on corticomotor excitability, primary studies evaluating responses to short‐lived experimental or acute clinical pain remain scarce. Cross‐sectional studies do not allow conclusions to be made regarding causality because they provide data on only a single time point, offering no information regarding corticomotor activity at ‘baseline’ or prior to pain onset (Bethlehem 1999). Without more longitudinal studies, it remains difficult to discern whether differences between people with and without lower limb pain are due to pain itself or some other factor. Finally, few studies have explored the effect of lower limb pain on corticomotor representations beyond the lower limb. This is an important step in determining whether lower limb pain is associated with localized or diffuse adaptations to corticomotor pathways. Though this review provides preliminary evidence that lower limb pain may be associated with changes in motor thresholds taken from remote muscles, further work in this space is required given the limited number of studies currently available.

While this review employed a rigorous approach towards data retrieval and analyses, certain limitations must be acknowledged. One such limitation is that database searches were restricted to full‐text articles, introducing the possibility of publication bias. This is because grey literature, such as unpublished reports, conference proceedings, and dissertations, was excluded, meaning that potentially relevant findings may have been overlooked. Another potential limitation of this review is that only English‐language literature was included in the analysis, which could limit the generalizability of the findings to non‐English‐speaking populations.

In conclusion, this systematic review found no overall effects of lower limb pain on MEP amplitudes recorded over the motor cortical hotspot. However, findings from other assessments of corticomotor excitability provide preliminary evidence that corticomotor responses to lower limb pain may be region‐ and diagnosis‐specific. Results from TMS mapping studies revealed shifts in CoG for representations of painful lower limb muscles, potentially underlying the loss of individuated muscle control observed in chronic lower limb pain. The data explored in this review also provide insight into the temporal profile of corticomotor responses to lower limb pain, with early experimental and acute pain‐inducing changes to map volume and motor threshold that differ from both those observed in experimental upper limb pain and chronic lower limb pain. Such findings suggest that corticomotor responses to lower limb pain may change over time, potentially presenting an opportunity to intervene and reduce risk of chronicity by “normalizing” excitability during acute pain. However, these interpretations must be considered with caution, as there remains a paucity of primary research exploring the effects of lower limb pain on corticomotor excitability. Further work involving experimental or acute clinical pain, in particular, is required to better probe causality and explore the corticomotor mechanisms underlying the transition from acute to chronic lower limb pain.

## Author Contributions

**Simon J. Summers**: conceptualization, methodology, writing – review and editing, investigation, data curation, supervision. **Jawwad Imam**: methodology, investigation, writing – original draft, writing – review and editing, formal analysis. **Edward Gray**: investigation, writing – review and editing, formal analysis. **Ariane Suhood**: methodology, investigation, writing – review and editing, formal analysis, data curation. **Ebonie Rio**: writing – review and editing. **Cherylea J. Browne**: Formal analysis, Writing – review and editing. **Nadia Moukhaiber**: writing – review and editing. **Rocco Cavaleri**: conceptualization, methodology, investigation, writing – review and editing, data curation, supervision, resources, project administration, formal analysis.

## Peer Review

The peer review history for this article is available at https://publons.com/publon/10.1002/brb3.70838

## Data Availability

Data that support the findings of this study are available from the corresponding author, Rocco Cavaleri, upon reasonable request.
